# Changes in the olfactory tract of patients with early Parkinson’s disease: A DTI tractography study

**DOI:** 10.1016/j.prdoa.2025.100396

**Published:** 2025-09-15

**Authors:** Pasquale Nigro, Andrea Chiappiniello, Filippo Bucherini, Pietro Chiarini, Alessandro Mechelli, Carlo Maremmani, Federico Paolini Paoletti, Roberto Tarducci, Andrea Fiacca, Lucilla Parnetti, Nicola Tambasco

**Affiliations:** a Movement Disorders Center, Neurology Department, Perugia General Hospital and University Hospital of Perugia, Perugia, Italy; b Department of Medical Physics, Perugia General Hospital, Perugia, Italy; c Department of Neuroradiology, Perugia General Hospital, Perugia, Italy; d Neurology Unit, Ospedale Apuane, Azienda USL Toscana Nord Ovest, Massa, Italy; e Neurology Department, Perugia General Hospital and University Hospital of Perugia, Perugia, Italy

**Keywords:** Magnetic resonance imaging, Diffusion tensor, MRI, Tractography, Parkinson’s disease, Hyposmia, Olfaction, Olfactory tract

## Abstract

**Introduction:**

olfactory impairment is a clinical feature of Parkinson’s disease (PD) even in the early phases. Diffusion tensor imaging fiber tracking analysis (DTI-FTA) has been able to highlight changes in the olfactory tract in PD, however in early phases of PD this analysis has not been investigated. We investigated the olfactory tract by DTI-FTA in early stages of PD (ePD).

**Methods:**

All patients were assessed using the Italian Olfactory Identification Test (IOIT), the Movement Disorder Society-Unified Parkinson’s Disease Rating Scale-Part III (MDS-UPDRS-III) and the Hoehn and Yahr (H&Y) scale. Diffusion imaging was conducted by 3 T MRI.

**Results:**

Overall, 26 PD patients and 20 healthy subject were recruited. Hyposmia was observed in all PD patients. DTI-FTA of the olfactory tract showed significant mean diffusivity (MD) increases and a tract volume decrease for PD group. MD and age, only in the PD group, were significant for multiple correlations.

**Conclusion:**

DTI-FTA identified microstructural changes in the olfactory tract even in hyposmic ePD patients.

## Highlights

• olfactory impairment is one of the most common and earliest symptoms in PD.
• changes in the olfactory tract in advanced PD have been demonstrated by DTI-FTA.
• we observed DTI-FTA changes in the olfactory tract even in the early phase of PD.
• this technique might provide the basis for developing an early biomarker for PD.

## 1 Introduction

Parkinson’s disease (PD) is a progressive neurological disorder characterized not only by motor symptoms but also by a wide range of non-motor manifestations, both of which can significantly impair daily functioning and reduce overall quality of life (QoL). A key therapeutic goal is to halt or slow disease progression through early intervention. Achieving this objective relies on the identification of specific and reliable biomarkers. However, no definitive biomarker has yet been established for early stages of PD (ePD).

Olfactory impairment is one of the most common and earliest non-motor symptoms. In fact, seventy-five to ninety percent of PD patients have been reported to present hyposmia which can profoundly deteriorate the QoL [1]. Moreover, idiopathic olfactory impairment has been associated with a high predictive value for the development of PD [2]; hyposmia often precedes motor symptoms by several years, and in 2019 it was included in the MDS Research Criteria for Prodromal PD [3]. For all these reasons, alterations in the olfactory system offer promising avenues for identifying potential biomarkers of the ePD.

The pathophysiology of olfactory dysfunction in PD is recognized as a complex phenomenon and poorly understood: postmortem studies have revealed pathological changes in brain regions associated with olfaction, including the olfactory bulb and tract, anterior olfactory nucleus, piriform cortex, amygdaloid complex, entorhinal cortex, as well as the hippocampus [4]. A predetermined sequence, rather than a random process, has been hypothesized to lead to neuronal damage, with the olfactory bulb being one of the first structures affected.

To date, odor identification tests have demonstrated high sensitivity but relatively low specificity. Therefore, a more comprehensive characterization of hyposmia in PD is essential to better understand its diagnostic and clinical significance. To this regard, previous studies have suggested that Diffusion Tensor Imaging (DTI) is sensitive in capturing early disease-associated changes in olfactory regions [[5], [6], [7], [8]]. Reduced fractional anisotropy (FA) in the central olfactory system was observed using Tract Based Spatial Statistics (TBSS) [9]. Moreover, DTI and statistical modeling have been reported to evidence that the olfactory regions can be effective in distinguishing de novo drug-naïve PD patients from healthy controls (HCs) [10]. Furthermore, using graph-theoretical and network-based analyses, Ming-Ching Wen and colleagues reported impaired connectivity of the right medial orbitofrontal cortex and the left gyrus rectus in ePD [11].

However, Atkinson-Clement et al., in their metanalytic study, emphasized the potential of DTI of the olfactory tract (OT) as a possible biomarker for ePD [12]. Notably, significant changes in anterior olfactory structures have been observed through both region-of-interest (ROI) analysis and TBSS [13,14]. Unlike traditional voxel-wise metrics, tractography enables the reconstruction of three-dimensional white matter fiber bundles. This technique allows for an accurate depiction of specific white matter pathways. Using DTI fiber tracking analysis (DTI-FTA), specific changes associated with OT degeneration in PD have been reported by our research team [15]. However, it remains unknown whether such alterations can also be detected in the early stages of the disease. To date, no study has specifically addressed this question.

Given the above, we investigated for any detectable PD-related DTI-FTA changes in the OT of drug-naive early-stage patients, aiming to identify potential markers of ePD.

## 2 Methodology

Overall, twenty-six ePD patients, defined according to the MDS Criteria for Clinically Established Early PD [16], were recruited from the Movement Disorders Unit, Perugia General Hospital, Italy. Throughout the study, all patients were drug-naïve. Additionally, twenty age-matched HCs were recruited from volunteers. None of the HCs referred a history of, or exhibited signs of, neurological disorders, and all denied any olfactory dysfunction. IOIT in HCs was not performed because idiopathic hyposmia is an uncommon condition in the general population. Moreover, for all patients, motor function was assessed using the Movement Disorder Society-Unified Parkinson's Disease Rating Scale - Part III (MDS-UPDRS-III), and disease stages were classified according to the Hoehn and Yahr (H&Y) scale.

Exclusion criteria included: inflammatory processes affecting the airways, MMSE score ≤24, history of head trauma, maxillo-facial surgery, nasal fracture, nasal polyps, and expansive lesions in the anterior cranial fossa.

### 2.1 Olfactory test

The odor identification ability of each PD patient was assessed, prior to undergoing magnetic resonance imaging (MRI), utilizing the Italian Olfactory Identification Test (IOIT). IOIT is a multiple-choice test, consisting of 33 odorants familiar to the italian population. Primarily for screening PD patients, this test has been utilized in Italy [17].

### 2.2 MRI acquisition protocol

PD patients and HCs were investigated using a 3 T Philips Achieva MR scanner, with an 8-channel head coil. MRI protocol included anatomical Fluid-Attenuated Inversion Recovery (FLAIR), T1-weighted, and T2-weighted sequences. Diffusion-weighted (DW) data were acquired using a single-shot spin-echo echo-planar imaging sequence across 32 non-collinear diffusion directions with a b-factor of 1000 s/mm^2^, along with a non-DW volume (acquisition parallel to the ethmoidal plane, FOV = 184 × 184 mm^2^, voxel size = 1.8 × 1.8 × 1.8 mm^3^, number of slices = 45 without slice gap, TE = 55 ms, TR = 9700 ms, flip angle = 90 deg, SPIR fat suppression, full k-space, 2 averaged acquisitions). TE was kept short by using maximum gradient strength/slew rate, parallel imaging, and partial Fourier encoding, whereas the 2 acquisitions increased the signal-to-noise ratio in the averaged image but doubled scanning time. To reduce DTI acquisition time to about 12 min, parallel imaging (SENSE 2) was used and only a cranio-caudal thickness of ≈8 cm was acquired, focusing on the OT.

### 2.3 DTI data processing and analyses

Dicom data were converted into nifti format using dcm2nii (https://www.mccauslandcenter.sc.edu/mricro/mricron/dcm2nii.html, output format FSL/SPM8—4D NIFTI).

Pre-processing of DW images was performed with the default protocol of DTIPrep (v. 1.2.11), which automatically corrected for eddy current distortions and head motion by removing low-quality directions and reorienting the b-matrix. Afterwards, Diffusion Toolkit (v. 0.6.4.1) was used to estimate the diffusion tensors and to perform deterministic tractography utilizing the FACT propagation algorithm (angle threshold = 30 deg, minimum FA = 0.05, minimum track length = 1 mm) and applying a spline filter.

**Fig. 1:**
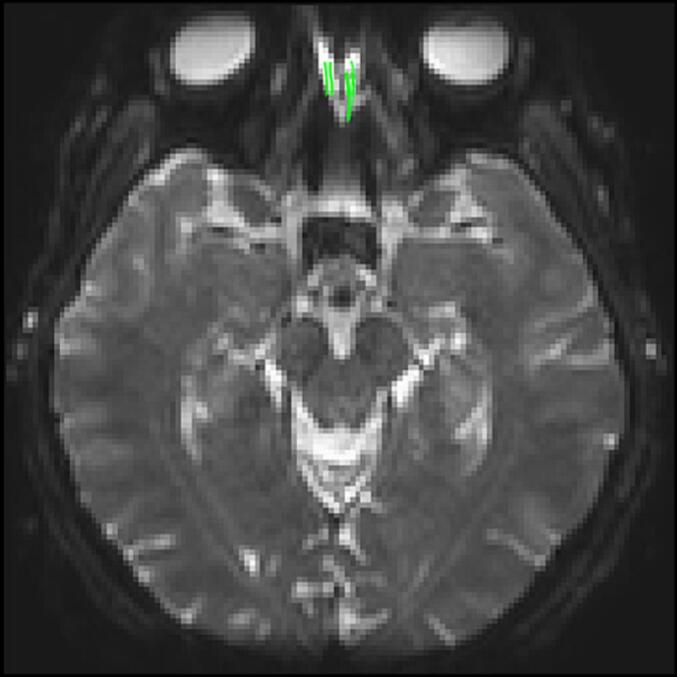
Axial slice of the non-diffusion-weighted volume acquired along with the DWdata where the olfactory bulb region is represented. Virtual manual dissections of the olfactory tracts obtained on a control subject using TrackVis.

Virtual manual dissections of the OT were performed, twice for each subject, with TrackVis (v. 0.6.1) **(**Fig. 1**)** by a tractographer, blinded to the clinical status of subjects. A voxel-based ROI was positioned in the OT distal region (including the olfactory bulbs), identified on a plane parallel to the ethmoidal plane. NOT-gated ROIs were eventually used to rule out any spurious reconstructed tracts. Afterwards, the estimates of the overall tract volume (TV), FA, mean diffusivity (MD), radial diffusivity (RD), and axial diffusivity (AD) were registered for each subject and averaged across the two dissections.

### 2.4 Statistical analysis

Statistical analysis was performed using Matlab R2024a. Descriptive statistics are reported as means and standard deviations for continuous variables and absolute frequencies and percentages for categorical variables. Intra-rater agreement was achieved for track volumes through intra-class correlation coefficient (ICC). The normality of each variable within the HCs and PD patients was assessed using the Shapiro-Wilk test. Since at least one group violated the normality assumption for the tested DTI parameters, two-group differences were evaluated using the nonparametric Mann-Whitney *U* test. To reduce bias related to the overall diffusivity, AD and RD were normalized by dividing them by MD. The resulting correlations were calculated using Spearman’s coefficients. All the statistical tests performed were adjusted for multiple comparisons using the Holm-Bonferroni method. For all analyses, a P value ≤5 % was considered statistically significant.

## 3 Results

### 3.1 Clinical variables

Demographic and clinical data of the studied cohorts are listed in Supplementary Table S1.

The PD group included 26 subjects, mean age 60.75 ± 5.90 years. The disease duration was 1.3 ± 0.6 years, the recorded H&Y stage score was 1.8 ± 0.4, while the MDS-UPDRS-III score was found to be 25.8 ± 9.2. Hyposmia was observed in all PD patients; the average IOIT score was 15.1 ± 4.3, ranging from 9 to 25 errors.

The HCs group which consisted of 20 subjects, had a mean age of 58.9 ± 10.6 years. No statistically significant differences in age and gender were observed between the two groups.

### 3.2 Analysis of processed DTI data

Overall, no significant disparity was observed for the two dissections for each subject, as indicated by the ICC for track volumes: 0.984 (P < 0.001).

The assessment of two-group differences in DTI-FTA of the OT revealed a significant MD increase and a TV decrease for the PD cohort compared with the controls (P < 0.05). No FA significant differences between the two groups were found. Similarly, no significant differences in AD and RD were observed after normalization for the corresponding MD value (Table 1).

**Table 1:** DTI-FTA of the olfactory tract (mean ± SD).

TV (ml) FA MD (mm^2^/s) AD/MD (mm^2^/s) RD/MD (mm^2^/s)	**Controls** 0.73 ± 0.30 0.146 ± 0.032 0.00149 ± 0.00025 1.15 ± 0.04 0.92 ± 0.02	**PD patients** 0.52 ± 0.23 0.134 ± 0.023 0.00171 ± 0.00036 1.16 ± 0.03 0.92 ± 0.02	**P value** 0.023 0.27 0.015 0.41 0.41

DTI-FTA: diffusion tensor imaging fiber tracking analysis; TV: tract volume; FA: fractional anisotropy; MD: mean diffusivity; AD: axial diffusivity; RD: radial diffusivity.

Spearman's rank correlation analysis indicated a significant association between MD and age was observed exclusively in the PD group (r = 0.52, P < 0.05) **(**Supplementary Fig. S21**)**. No significant correlations were found between AD/FA/MD/RD/TV values and parameters for motor/olfactory functions.

## 4 Discussion

The DTI-FTA of the OT resulted being both feasible and reliable and was able to detect structural differences between hyposmic ePD patients and HCs. From our investigation, the most significant finding was the difference between the two group distributions for DTI values, exhibiting a significant MD increase and a significant TV decrease in the PD group. These findings in a previous published 7 T-MR study had not been observed [18], probably due to the higher MR field, which is known to induce artifacts. Likewise, we employed a 3 T MRI scanner and, in order to reduce susceptibility artifacts an optimized single-shot DW-EPI sequence, tailored for examining the OT, was used. Moreover, to increase the signal-to-noise ratio, two acquisitions were collected.

The same methodology used in the present study, was used in a previous investigation [15]. However, we did not investigate ePD patients, instead the cohort consisted of patients with moderate PD. In both studies, a TV decrease was observed in the PD group. On the other hand, compared with our previous work, in the present study significant differences between PD patients and HCs were found for MD and not for FA. MD, by reflecting the total magnitude of diffusion and hence providing information regarding any alterations in the extracellular volume, might be able to detect neurodegenerative processes in the early stage, not evidenced by FA. Interestingly, Scherfler et al. and colleagues had reported an increased MD in the OT of PD patients [19].

Additionally, in agreement to previous results from literature [18], in our study we observed significant positive correlations between MD values and age in the PD group. Since this correlation was observed only in the PD group, we hypothesize that this result may have been related to underlying neurodegenerative processes. In fact, a significant correlation between olfactory impairment and advanced age in PD patients has been demonstrated [19].

On the other hand, as in previous reports [20], we did not observe any correlations between the severity of the motor dysfunction and DTI values of the OT. Thereby, contributing to the hypothesis that the progression of motor impairments and the neuropathological processes within the anterior olfactory systems might have distinct and divergent courses of time. If proven true, this could provide valuable information in the clinical setting. Specifically, it could lead to a better understanding of the processes underlying the ePD.

DTI parameters show great potential for assessing axonal and myelin sheath integrity. [[10], [21]] At present, the pathological interpretation of FA reduction in neurodegenerative diseases is not clear and can be attributed to a number of factors including edema, demyelination, gliosis, and inflammation. A study using a mouse model of PD following 1-methyl-4-phenyl1-1,2,3,6-tetrahydropyridine (MPTP) intoxication demonstrated that cell loss in the pathologically involved brain regions reflects FA reductions in the WM projections of those areas. According to Ibarretxe-Bilbao et al, the reduced values found in the hyposmic early PD patients could indicate cell loss in the olfactory bulb and loss of axonal projections to primary olfactory areas [4].

The above findings lend support to Braak's hypothesis, which proposes an early involvement of the olfactory bulbs and tracts in PD pathology [4]. They also align with theories identifying the olfactory bulb as a gateway for pathogens or environmental insults, potentially triggering the spread of pathological changes throughout the brain.

The major limitations of our study include the low number of investigated patients and HCs, which limits the statistical power and increases the risk of type I/II errors, along with the indistinguishability of our cohorts along two axes, namely, PD vs HCs and hyposmic vs normosmic, making it impossible to reliably attribute change to one investigated cause or another. Furthermore, TV alone provides less meaningful insights compared to volumetric measurements of the olfactory tracts using 3D-T1 imaging. Lastly, given the single-center nature of this study, the generalizability of the results is limited and should be confirmed through validation in independent, multi-center cohorts.

In conclusion, DTI-FTA appeared to be able to identify microstructural changes in the OT of the hyposmic ePD patients. Therefore, this novel technique holds promise for accurately characterizing hyposmia in ePD, potentially improving diagnostic accuracy and providing the basis for developing an early biomarker. The impact of validating this finding in large multicenter studies of ePD patients, would be substantial, facilitating more effective identification and screening of patients at an early stage.

## CRediT authorship contribution statement

**Pasquale Nigro:** Writing – original draft, Data curation, Conceptualization. **Andrea Chiappiniello:** Writing – original draft, Methodology, Formal analysis. **Filippo Bucherini:** Investigation, Formal analysis, Data curation. **Pietro Chiarini:** Investigation, Data curation. **Alessandro Mechelli:** Methodology, Data curation. **Carlo Maremmani:** Resources, Data curation. **Federico Paolini Paoletti:** Data curation. **Roberto Tarducci:** Supervision, Software. **Andrea Fiacca:** Methodology. **Lucilla Parnetti:** Writing – review & editing, Supervision, Conceptualization. **Nicola Tambasco:** Writing – review & editing, Supervision, Conceptualization.

## Declaration of competing interest

The authors declare that they have no known competing financial interests or personal relationships that could have appeared to influence the work reported in this paper.
