# Supplementary figures and images for: Changes in the olfactory tract of patients with early Parkinson’s disease: A DTI tractography study
Source: Clin Park Relat Disord. 2025 Sep 15;13:100396. doi: 10.1016/j.prdoa.2025.100396 (PMC12489904; doi:10.1016/j.prdoa.2025.100396)

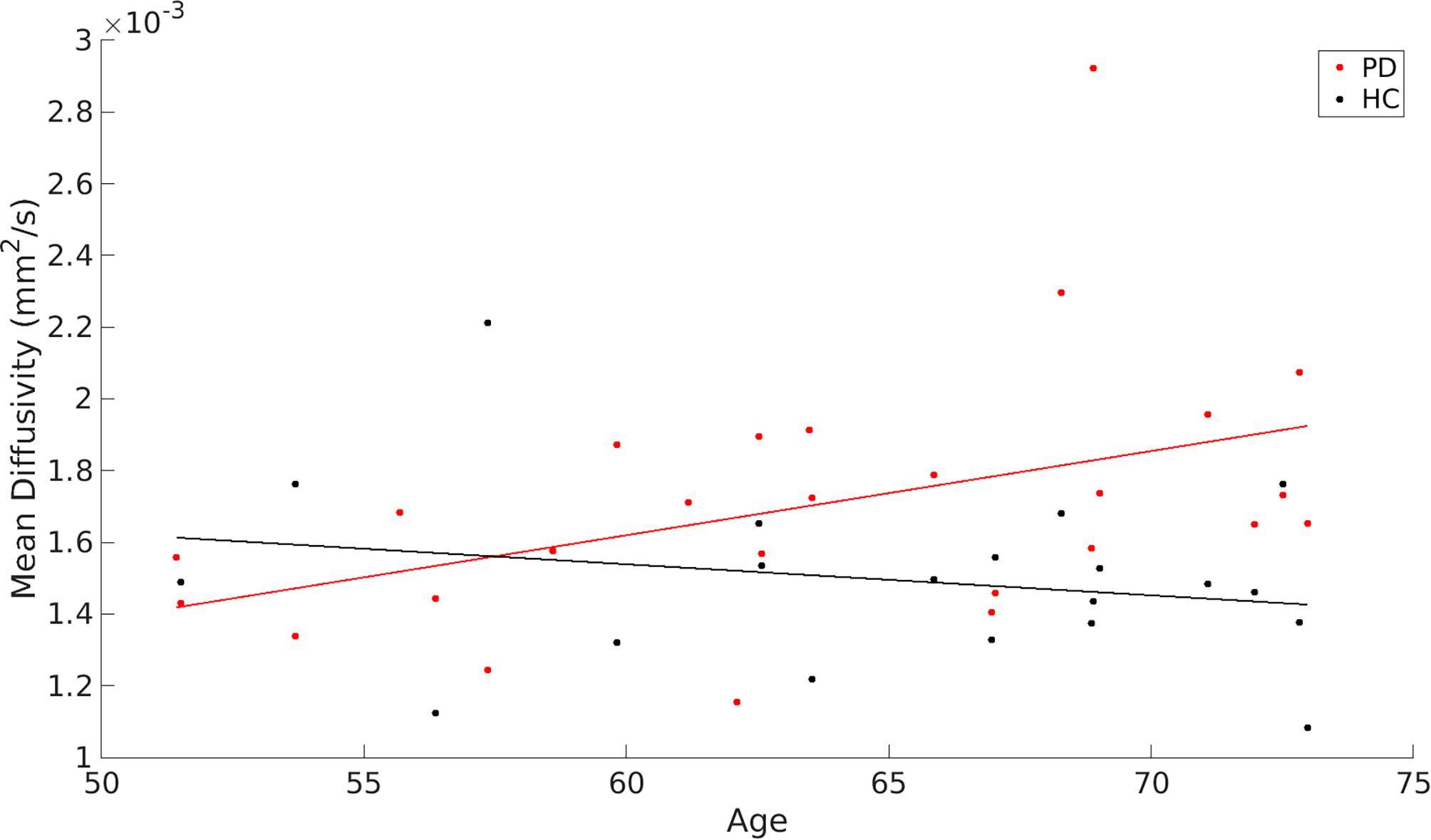

Supplement: Supplementary Fig. 1 [file mmc1.jpg]
