# Supplementary material for: Changes in the olfactory tract of patients with early Parkinson’s disease: A DTI tractography study
Source: Clin Park Relat Disord. 2025 Sep 15;13:100396. doi: 10.1016/j.prdoa.2025.100396 (PMC12489904; doi:10.1016/j.prdoa.2025.100396)
Supplement: Supplementary Data 2 [file mmc2.docx]

**Supplementary Information**

**Supplementary Table S1.**

Characteristics of the studied population (mean±SD).

| Male/female, no.  Age, yr  Duration of disease, yr  H&Y  MDS-UPDRS III  IOIT score | **PD patients (n=26)**  17/9  60.8±5.9  1.3±0.6  1.8±0.4  25.8±9.3  15.1±4.3 | **Controls (n=20)**  12/8  58.9±10.6 |
| --- | --- | --- |

**Supplementary FIG. S1.**

Comparison of olfactory tract mean diffusivity (left panel) and tract volume (right panel) between healthy controls (HCs) and patients with Parkinson’s disease (PD)

**Supplementary FIG. S2.**

Correlation between mean diffusivity values and age in PD patients and in HCs group. A significant correlation, only for PD patients, was found (r=0.52, P<0.05, Spearman’s rank correlation)
